# Dissolution-permeation approach for biopharmaceutical evaluation: a feasibility study using naproxen

**DOI:** 10.3389/jpps.2026.16570

**Published:** 2026-05-11

**Authors:** Andrejs Sitovs, Katarina Gurkina, Liga Petersone, Valentyn Mohylyuk

**Affiliations:** 1 Leading Research Group, Faculty of Pharmacy, Rīga Stradiņš University, Riga, Latvia; 2 Department of Pharmacology and Pharmacotherapy, Rīga Stradiņš University, Riga, Latvia

**Keywords:** BCS, dissolution, naproxen, permeability, permeation

## Abstract

**Objectives:**

Dissolution and/or release profile alone are insufficient to predict the *in vivo* absorption of poorly soluble drugs. Thus, permeation test becomes a critical component for biopharmaceutical assessment. Currently, none of the dissolution-permeation systems include the compendial dissolution, or provide large acceptor volumes, or are compatible with *ex vivo* membranes. Addressing the limitations of the existing dissolution-permeation testing systems, we propose the integration of the Ussing (permeability) chamber to the dissolution apparatus.

**Methods:**

The assembled dissolution-permeation system evaluated the effect of 5% and 10% soy L-α-phosphatidylcholine in dodecane (LiDo) and permeable membrane material on apparent permeability coefficients of naproxen, a BCS II class drug.

**Results:**

Naproxen release from the tablets reached approximately 100% within 30 min. Naproxen P_app_ across a 0.45 µm PVDF membrane was 0.91 ± 0.35 × 10^-8^ cm/s for 5% LiDo, and 0.75 ± 0.23 × 10^-8^ cm/s for 10% LiDo. The 0.20 μm PC membrane with 5% LiDo showed a P_app_ of 1.99 ± 0.57 × 10^-8^ cm/s.

**Conclusion:**

The proposed compendial dissolution-permeation system with an Ussing chamber allowed for the simultaneous determination of dissolution and permeability of naproxen. All P_app_ values obtained were approximately 100-fold lower than those reported in the literature. The results may have been influenced by the differences in sink conditions and permeable membrane composition. The use of a PC membrane resulted in higher permeability, compared to the PVDF membrane. Adequately improved, this methodology could be transferred for the *ex vivo* membrane dissolution-permeability testing.

## Graphical Abstract

**Figure:**
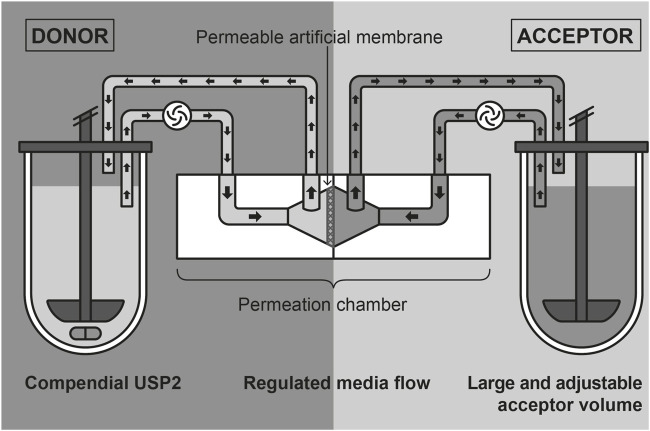

## Introduction

According to the Biopharmaceutical Classification System (BCS) and its modification, the Developability Classification System (DCS), orally administered drugs are classified into four classes based on their permeability across the gastrointestinal (GIT) membranes and dose solubility in relevant GIT fluid volumes [1]. The modern paradigm in pharmaceutical science pipelines clearly shows the shift from drugs with no solubility issues in aqueous media (BCS classes I/III) to the predominance of poorly soluble drugs (BCS classes II/IV) [2]. For the latter, the dissolution and/or release profile data alone are insufficient to predict *in vivo* drug behaviour, making permeation testing a critical component of biopharmaceutical assessment [3]. Permeability across the GIT membranes, which determines the absorption and influences the bioavailability, is crucial for assessing the intrinsic properties of drug substances and for formulation screening. Currently, dissolution-permeation testing is mostly applied to poorly soluble drugs. Few systems are commercially available, each with advantages and limitations, which have been addressed comprehensively in the recent review article regarding dissolution-permeation systems [3].

To the best of our knowledge, the volume of the acceptor compartment of the commercially available dissolution-permeation systems, which includes compendial dissolution/release testing does not exceed 50 mL. None of the commercially available dissolution-permeation systems allows simultaneous compatibility with *ex vivo* membranes. Addressing the limitations of the existing dissolution-permeation testing systems, we propose the integration of the Ussing (permeability) chamber to the compendial dissolution apparatus. Dissolution testing procedures and volumes are highly standardised, and they allow the use of larger acceptor compartment volumes, which is crucial for poorly soluble compounds, whereas the Ussing chamber is compatible with *ex vivo* tissue and artificial membranes. Compared to the equipment, such as MicroFLUX and MacroFLUX, the proposed system could be compatible with an excised intestinal tissue. Compared to the parallel artificial membrane permeability assay (PAMPA), and equipment, such as MicroFLUX and MacroFLUX, the proposed system provides larger and adjustable acceptor compartment [3]. The innovative compendial dissolution-permeation method, when applied in biopharmaceutical research of oral dosage forms, targets multiple current global pharmaceutical drug development problems such as, the improvement of the bioavailability of oral formulations, the reduction of financial investments in clinical studies, the increase in success rate and reduction in risks of clinical studies, the improvement in therapeutical outcomes of drug treatment, the reduction of the drug doses required to achieve therapeutical effects, the reduction of non-absorbed drug amount and its impact on the environment.

Primarily, this study aimed to assemble a dissolution-permeation system setup based on the compendial dissolution (USP2) apparatus and the commercially available Ussing chamber. A secondary aim was to utilise the assembled setup and evaluate the effect of 5% and 10% soy L-α-phosphatidylcholine in dodecane (LiDo) and permeable membrane material on the apparent permeability coefficients (P_app_) of naproxen. Naproxen is a passively transported drug, and although it exhibits limited solubility in acidic biological fluids, it exhibits high solubility in close to neutral pH solutions [4], with no issues regarding permeability across intestinal membranes. Lastly, the observed naproxen permeability was compared with literature data.

## Methods

### Chemicals and reagents

Naproxen of Ph. Eur. purity standard (Sigma Aldrich, USA), and commercially available coated tablets Nalgesin (KRKA, Slovenia) containing 550 mg naproxen sodium, equivalent to 500 mg naproxen were used. KH_2_PO_4_ and NaOH were obtained from Sigma Aldrich, USA. Soy L-α-phosphatidylcholine (95%) was obtained from Avanti Polar Lipids, USA. Anhydrous dodecane (>99%) was obtained from Sigma Aldrich, USA. Ultra-purified water was obtained with a StakPure system (StakPure, Germany). 0.45 µm Hydrophobic PVDF (Durapore) membrane filters (thickness of 125 µm) were obtained from Merck Millipore (Millipore, Ireland). 0.2 µm polycarbonate membrane filters (PC, thickness of 10 µm) were obtained from GVS (USA).

### Dissolution-permeation system setup

**FIGURE 1:**
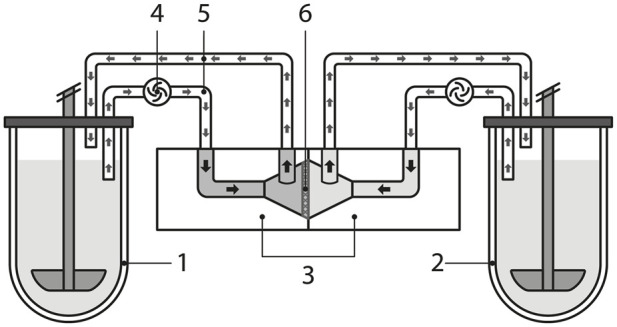
Schematic representation of the dissolution-permeation system. 1 – donor vessel, 2 – acceptor vessel, 3 – Ussing chamber (left–donor compartment, right–acceptor compartment), 4 – peristaltic pump, 5 – silicone tubing, 6 – permeable membrane.

The setup (Figure 1) consisted of a compendial USP2 dissolution apparatus (Double ATSXstend, Sotax, Switzerland), a 6-channel peristaltic pump (LabN1-II/AMC6, Shenchen, China) with silicone tubing, and a CHM1 Ussing chamber with a 1.13 cm^2^ membrane opening (World Precision Instruments, USA). Two USP2 vessels were used simultaneously with a single Ussing chamber: one vessel contained the dissolution media with the permeable drug (donor compartment), and the other was filled with the acceptor media (acceptor compartment). The Ussing chamber was mounted to an in-house built stand and secured with screws to prevent media leakage. Silicone tubing (FedroTek, China) with an internal diameter of 2 mm and a wall thickness of 1 mm was securely connected to the Ussing chamber with original Luer-type fittings (World Precision Instruments, USA), ensuring a leak-free system. Each tube was 1 m in length. The withdrawing ends of the tubes were placed through the dissolution apparatus apertures into the media, approximately 1 cm below the media surface, and connected to the peristaltic pump. The returning tube passed through the same apertures, allowing the media to drip passively onto the surface of the USP2 vessels from a height of approximately 1 cm above the media, maintaining a closed circulation system. The dissolution apparatus apertures were covered with adhesive tape to minimise media loss due to evaporation. The water bath temperature was maintained at 37 °C, the paddle rotation speed was set to 50 rpm, and the media pumping flow rate was 6 mL/min.

### Naproxen dissolution-permeation study

Nalgesin 550 mg tablets were used in both dissolution and dissolution-permeation studies. The dissolution study that used Nalgesin 550 mg tablets (n = 8) was performed in the compendial USP2 dissolution apparatus (Double ATSXstend, Sotax, Switzerland). The dissolution media was pharmacopoeial phosphate buffer solution (PBS, 1,000 mL, pH 6.8, KH_2_PO_4_, NaOH). The water bath temperature was maintained at 37 °C with a paddle rotation speed of 50 rpm. Sampling was performed after 5, and then after every 10 min over a duration of 120 min.

Naproxen standard and Nalgesin 550 mg tablets were dissolved in 1,000 mL PBS with a pH of 6.8 and were used in the donor vessel of the dissolution-permeation study. The acceptor vessel contained 1,000 mL PBS with a pH of 7.4 (KH_2_PO_4_, NaOH). The paddle rotation speed was 50 rpm.

A permeable membrane filter (0.45 µm hydrophobic PVDF, 0.2 μm PC) was mounted in the Ussing chamber and covered with 18.4 µL of soy L-α-phosphatidylcholine solution in dodecane (LiDo, 5% or 10% w/v). Permeated naproxen in the acceptor media was UV-quantified on-line with a sampling time of 15 min over a total duration of 180 min. All experiments were performed at least in triplicates. The apparent permeability coefficients (P_app_) were calculated from the values on the linear part of the naproxen concentration-time curve in the acceptor compartment. Only the concentration points above the lower limit of quantification (LOQ) were accounted. The concentration points above the LOQ corresponded to the end of the lag time (lag time concentration-time points were omitted). Linear correlation was calculated in accordance to the following equation:

$$Correl\left(x,y\right)=\frac{\sum \left(x-\bar{x}\right)\left(y-\bar{y}\right)}{\sqrt{\sum {\left(x-\bar{x}\right)}^{2}\sum {\left(y-\bar{y}\right)}^{2}}}$$

where, x and y are the sample means (average) array 1 and (average) array 2. For this purpose, MS Excel (Microsoft 365; Redmond, Washington, DC, USA) was used.

The curves with correlation coefficient (R^2^) values equal to or higher than 0.99 were considered to be linear. The P_app_ was calculated using the following equation:

$$P_{app}=\frac{dQ/dt}{A\times C_{0}}$$

where:

*dQ*/*dt*–rate of naproxen appearance in the acceptor medium (µg/mL/s)
*A*–exposed permeable membrane area (cm^2^)
*C*_0_ – initial naproxen concentration in the donor medium (µg/mL)

The P_app_ values obtained were statistically compared in MS Excel. The impact of the concentration of LiDo (5% or 10% w/v) was evaluated using Single Factor Anova, and the impact of the membrane (PC or PVDF) was evaluated using t-Test: Two-Sample Assuming Unequal Variances.

The membrane filters were studied using scanning electron microscopy (SEM). SEM pictures were captured with a Hitachi S-4800 microscope (Hitachi High-Tech Corp., Japan) at 2.0 kV under vacuum to assess pore morphology and size.

### Quantification

The quantification of naproxen in the donor media (dissolution and dissolution-permeation study) was performed with an off-line method. At predetermined time points (5, 10 min, then every 10 min) 3 mL of the media was withdrawn, diluted 1:100 in PBS pH 6.8, and the absorbance was measured spectrophotometrically at an absorbance wavelength of 334 nm using a standalone Shimadzu UV-1900i spectrophotometer (Shimadzu, Japan). The calibration concentration range was 0.05–5.00 μg/mL. The calibration equation was C = (Abs +0.0016)/0.3601.

The quantification of naproxen in the acceptor media was performed on-line. At predetermined time points (every 15 min) 3 mL a small amount of the media was withdrawn, naproxen was quantified spectrophotometrically at an absorbance wavelength of 334 nm using a Specord 200 Plus spectrophotometer (Analytik Jena, Germany), and automatically returned to the vessel afterwards. The calibration concentration range was 0.01–0.50 μg/mL. The calibration equation was C = (Abs +0.0009)/0.3270.

## Results

The absorption wavelengths of naproxen were similar in both PBS pH 6.8 and 7.4, therefore the quantification was performed at 334 nm each time. The on-line and off-line quantification of naproxen were linear over the concentration ranges of 0.01–0.50 μg/mL (R^2^ ≥ 0.99) and 0.05 – 5.00 μg/mL (R^2^ ≥ 0.99) respectively. The lower limit of quantification was 0.03 μg/mL and the limit of detection was 0.01 μg/mL. The acceptor and donor media volume loss after the experiment was 0.3–1.3%.

**FIGURE 2:**
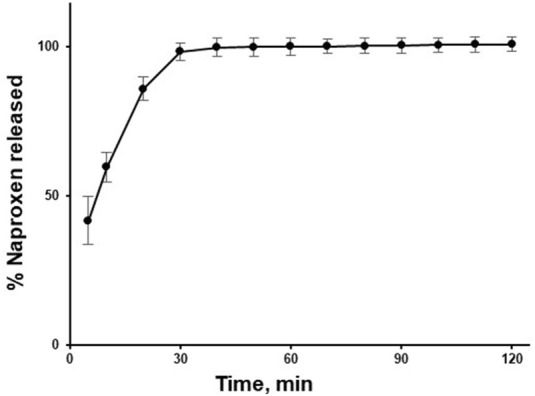
Average naproxen release profile from 550 mg naproxen sodium tablets (n = 8) in PBS pH 6.8.

The naproxen release from tablets exceeded 50% (corresponding to 250 mg naproxen per litre of donor media) within 10 min of dissolution test, and reached approximately 100% within 30 min (Figure 2).

**FIGURE 3:**
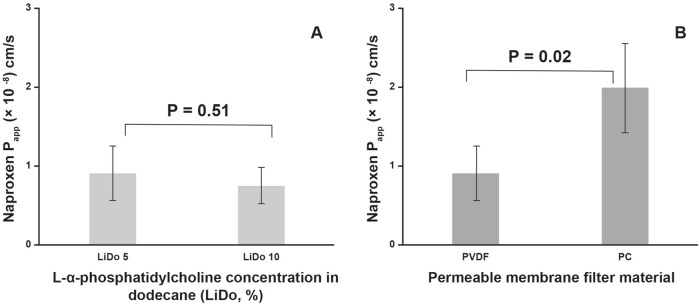
Panel **(A)**–comparison of phospholipid concentration in dodecane on naproxen apparent permeability across 0.45 µm PVDF membrane filter (LiDo 5: n = 5, LiDo 10: n = 7). Panel **(B)**–comparison of membrane filter material, containing 5% L-α-phosphatidylcholine in dodecane on naproxen apparent permeability (PVDF: n = 5, PC: n = 3). PVDF – 0.45 µm pore size hydrophobic polyvinylidene fluoride membrane, PC – 0.20 µm pore size polycarbonate track etched membrane.

The calculated P_app_ for naproxen permeability across a 0.45 µm PVDF membrane filter was 0.91 ± 0.35 × 10^-8^ cm/s with 5% LiDo, and 0.75 ± 0.23 × 10^-8^ cm/s with 10% LiDo. With a 0.20 μm PC membrane filter the P_app_ was 1.99 ± 0.57 × 10^-8^ cm/s with 5% LiDo (Figure 3). In the experiment using the same donor and acceptor media composition (PBS pH 6.8) across 10% LiDo on 0.45 µm PVDF, the average P_app_ of naproxen was 0.95 × 10^-8^ cm/s.

**FIGURE 4:**
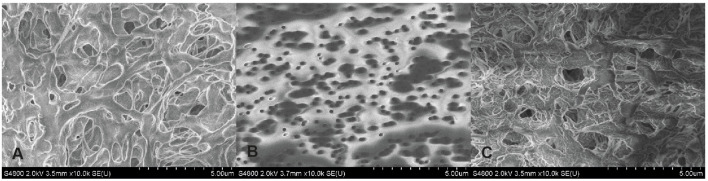
Scanning electron microscopy pictures of filter membranes. **(A)** PVDF – 0.45 µm pore size hydrophobic polyvinylidene fluoride membrane. **(B)** PC – 0.20 µm pore size polycarbonate track etched membrane. **(C)** Membrane disc used in the commercially available dissolution-absorption flux system MicroFLUX (for comparison) [5].

Microscopy confirmed differences in size and structure of the membrane filter pores (Figure 4).

## Discussion

Naproxen (pKa 4.2) exhibits limited solubility in acidic biological fluids and belongs to the BCS Class II drugs [6]. The studied dose of 550 mg naproxen sodium (500 mg naproxen) in 1,000 mL donor media was in line with required experimental sink conditions. Sink conditions were achieved, considering the reported solubility of naproxen at 37 °C to be 1.452 ± 0.004 mg/mL in PBS (pH 7.4) [4], and 3.619 ± 0.113 mg/mL in PBS (pH 6.8) [7], corresponding to sink indices of 2.9 and 7.2 for PBS pH 7.4 (acceptor media) and 6.8 (donor media), respectively [8]. At pH values corresponding to small intestine lumen (6.8–7.4) [9], over 99% of naproxen exists in ionised water-soluble form, according to the Henderson-Hasselbalch equation. Considering that naproxen sodium tablets used in the present study released the whole dose of naproxen in the salt form within the first 30 min of the study, their use in dissolution-permeation study was justified and considered equal to naproxen acid solution use.

The proposed dissolution-permeation system using a membrane similar to soy lecithin in dodecane in the PAMPA methodology [10] enabled the simultaneous determination of dissolution and permeability of naproxen. Despite the fact that artificial membranes have already been installed in the Ussing chamber [11], our setup proposed the interconnection of compendial dissolution testing apparatus with a permeation chamber. Two hydrophobic membrane-type filters were used as support for LiDo, similar to those used in PAMPA [10]. The hydrophobic PVDF membrane filters coated with LiDo are typically used in PAMPA [10], and reported to be used in an artificial membrane-Ussing model [11], while PC is another material used as a support membrane in PAMPA [10]. A LiDo volume of 18.4 µL was chosen to align with instructions for commercially available permeation flux system, which use 25 µL LiDo on a 1.54 cm^2^ filter membrane [5], and with a previously reported artificial membrane-Ussing model that used 20 µL of phospholipid solution [10]. The permeable area, meanwhile, was determined by the size of the Ussing chamber aperture.

All P_app_ values obtained in this study were significantly lower compared to those reported using the artificial membrane-Ussing model (19.55 ± 0.47 × 10^-6^ cm/s) [11] and with naproxen permeability obtained in PAMPA experiments 4.2 × 10^-6^ cm/s [12]. This 100-fold decrease in permeability can be attributed to the absence of chemical scavenger simulating serum proteins or any other solubility-enhancing additive, potentially affecting the flux across permeable membranes [13]. Nevertheless, considering the virtually complete ionisation of naproxen in PBS, sink conditions were established in the acceptor media.

Another reason for possible differences in the P_app_ values between our results and PAMPA is differences in LiDo composition. In the latter, 20% (w/v) dodecane solution of lecithin was used [12]. With increasing soy lecithin concentration from 10% to 74% in dodecane, the permeability of naproxen showed a tendency to decrease [10]. Thus, lower LiDo concentrations were chosen for this study. Conversely, no statistically significant difference was observed when using 5% or 10% LiDo with the 0.45 µm PVDF membrane. A statistically significant difference in naproxen P_app_ was observed across the polycarbonate membrane compared with the PVDF membrane in the presence of 5% LiDo. This difference may be explained by the size and structure of the membrane pores observed microscopically. Despite the pores of PVDF membrane are on average 2.25 times larger than of PC, they appear more complex, which can possibly affect the coating and distribution of LiDo on the membrane. SEM microscopy of the PVDF membrane used in this study has not revealed any differences in pore structure and size compared to the PVDF membrane included in the commercially available dissolution-absorption flux system (MicroFLUX). Notably, the thickness of the PVDF membrane is approximately 125 μm, but according to specifications, the thickness of the PC membrane is 10 µm.

The suggested dissolution-permeation setup with an artificial membrane and a lipid layer is applicable for simulation of the passive transport only. Thus, it is limiting the applicability of such approach for the testing of drugs with other drug transportation mechanisms across the intestinal membrane. Nevertheless, the same setup is compatible with *ex vivo* membranes and could be used for the dissolution-permeation assays of drugs with active and facilitated drug transport.

The proposed compendial system incorporating an Ussing chamber for the simultaneous determination of dissolution and permeability of naproxen, a BCS class II drug, was assembled. LiDo concentration (5 vs. 10%) did not appear to significantly influence naproxen permeability. The use of a permeable PC membrane resulted in higher permeability, compared to the PVDF membrane. Based on the positive result of the *in vitro* dissolution-permeation study, other poorly soluble drugs can be tested for permeability *in vitro*. Consequently, this methodology could be further applied to more physiologically relevant *ex vivo* membrane permeability testing incorporating active transport, performed in tandem with compendial dissolution and/or release testing of drugs or drug formulations.

## Data Availability

The raw data supporting the conclusions of this article will be made available by the authors, without undue reservation.
